# Patients’ perspective on prostatic artery embolization: A qualitative study

**DOI:** 10.1177/20503121211000908

**Published:** 2021-03-12

**Authors:** Alexander Holm, Hans Lindgren, Mats Bläckberg, Marika Augutis, Peter Jakobsson, Mattias Tell, Jonas Wallinder, Karl-Johan Lundström, Johan Styrke

**Affiliations:** 1 Department of Surgical and Perioperative Sciences, Urology and Andrology, Umeå University, Umeå, Sweden; 2 Department of Clinical Sciences, Faculty of Medicine, Lund University, Lund, Sweden; 3 Department of Interventional Radiology and Surgery, Helsingborg Hospital, Helsingborg, Sweden; 4 Department of Urology, Helsingborg Hospital, Helsingborg, Sweden; 5 Department of Research and Development, Sundsvalls Hospital, Sundsvall, Sweden; 6 Department of Radiology, Östersunds Hospital, Östersund, Sweden; 7 Department of Surgery, Sundsvalls Hospital, Sundsvall, Sweden

**Keywords:** Prostatic artery embolization, benign prostate hyperplacia, health care users’ experiences, doctor–patient, nurse–patient communication, patient education

## Abstract

**Objectives:**

The aim was to describe the patients’ experience of undergoing prostatic artery embolization.

**Methods:**

A retrospective qualitative interview study was undertaken with 15 patients of mean age 73 years who had undergone prostatic artery embolization with a median duration of 210 min at two medium sized hospitals in Sweden. The reasons for conducting prostatic artery embolization were clean intermittent catheterization (n = 4), lower urinary tract symptoms (n = 10) or haematuria (n = 1). Data were collected through individual, semi-structured telephone interviews 1–12 months after treatment and analysed using qualitative content analysis.

**Results:**

Four categories with sub-categories were formulated to describe the results: a diverse experience; ability to control the situation; resumption of everyday activities and range of opinions regarding efficacy of outcomes. Overall, the patients described the procedure as painless, easy and interesting and reported that while the procedure can be stressful, a calm atmosphere contributed to achieving a good experience. Limitations on access to reliable information before, during and after the procedure were highlighted as a major issue. Practical ideas for improving patient comfort during the procedure were suggested. Improved communications between treatment staff and patients were also highlighted. Most patients could resume everyday activities, some felt tired and bruising caused unnecessary worry for a few. Regarding functional outcome, some patients described substantial improvement in urine flow while others were satisfied with regaining undisturbed night sleep. Those with less effect were considering transurethral resection of the prostate as a future option. Self-enrolment to the treatment and long median operation time may have influenced the results.

**Conclusions:**

From the patients’ perspective, prostatic artery embolization is a well-tolerated method for treating benign prostate hyperplacia.

## Introduction

Lower urinary tract symptoms (LUTS) affect the Quality of Life (QoL) in 15% to 60% of men. Transurethral resection of the prostate (TURP) is currently regarded as the leading method in treating benign prostate hyperplacia (BPH).^1^ Prostatic artery embolization (PAE) has emerged as a new treatment option during recent years, but there are concerns regarding long-term efficacy.^2^ The method consists of an endovascular approach identifying and embolizing the prostatic arteries with polyvinyl alcohol spheres to inflict transformation of the prostate to a fibrous capsule.^3^

Patients’ perspectives on health care methods are important and can give new insights to how care can be improved. Studies within the field of endovascular surgery show that the need for information and the ability to cope with the healing process and side-effects are important issues for the patients and that there is potential for improvement of nursing care.^4,5^ It has also been shown from dental surgery and breast surgery that patients do not always have realistic expectations on what to expect from the pre- and postoperative phase.^6,7^ We hypothesized that looking into patients’ experiences of PAE using a qualitative method would give new information on how patients tolerate the method and how to improve care surrounding these procedures.

The primary aim of this study was to investigate the patient perspective of PAE in terms of communication and comfort. A secondary aim was to describe the patients’ satisfaction with the treatment outcomes. Participants who underwent PAE at the two treatment centres in the initial experience in Sweden 2016-2017 were invited to be interviewed.

## Materials and methods

### Participants

Twenty consecutive patients, treated with PAE at two medium-sized hospitals in Sweden between October 2016 and July 2017, were invited to an interview. Inclusion criteria: Swedish speaking males aged over 18 years, 1–12 months post-treatment. Non-consenting participants or those identified with dementia were excluded.

**Figure 1.**
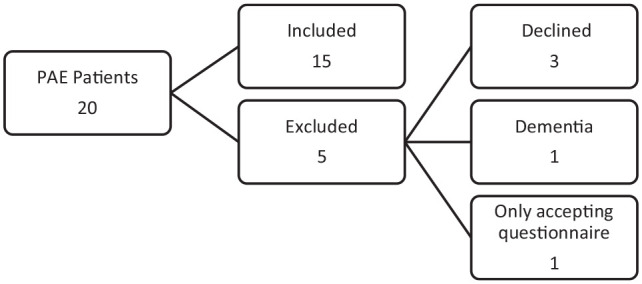
Sample selection. PAE: prostatic artery embolization.

Five patients were excluded and 15 included (Figure 1). The participants’ characteristics are shown in Table 1.

**Table 1.** Characteristics of study population.

	n (%)
ASA = 3 Indication	6 (40)
CIC	4 (27)
Haematuria/LUTS	1 (7)
LUTS	10 (67)
Unilateral technique	4 (27)
	median (IQR)
Age (years)	73 (68, 75)
Months since treatment	6 (2.5, 8.5)
Minutes in theatre	210 (180, 240)

ASA: American society of Anaesthesiologists physical status system; CIC: Clean Intermittent Catheterization; LUTS: Lower Urinary Tract Symptoms.

### Data collection

The patients received a letter with study information approximately 2 weeks before the interview. The telephone interviews were conducted by A.H., a male final year medical student who had no prior or later contact with the interviewees. The interviews were a part of the research methodology course at the medical school at Umeå University. A.H. has journalism education and is experienced in conducting interviews. The interview guide (see supplemental Appendix 1), with semi-structured questions, was subject to a pilot interview with one patient and evaluated by A.H., M.A., a senior qualitative researcher and lecturer in research methodology and J.S., lecturer in urology and consultant urologist, supervisor for AH.

Interviewees were advised about confidentiality and consent. After A.H. presented himself and the aim of the interviews, the opening question was ‘What was your experience of the procedure?’ Follow-up questions were asked to encourage participants to share their experiences. The questions included preoperative information, memories of the procedure, and the time after the procedure. The final question was ‘Would you recommend this to a friend and if so why?’ The interviews were conducted from September to October 2017 at a pre-booked appointment time. The interviews (median length 42 min (range: 21 to 64 min)) were recorded and transcribed verbatim. The transcripts were not returned to the participants for comments or correction. We noticed that after 10 interviews, most data had been previously discussed in the interviews and saturation was reached.

### Analysis

Based on qualitative theory,^8^ the interviews were analysed using content analysis as described by Graneheim and Lundman.^9^ Initially, A.H. read the transcripts to get a sense of the whole. Sentences were divided into meaning units and condensed into codes. The codes were grouped into subcategories and then categories. Throughout the process, A.H., M.A. and J.S. had regular meetings discussing the material. A seminar with researchers in qualitative method, urologists and a patient representative was conducted to further validate the analysis.^8^ The participants did not provide feedback on the findings.

### Ethics

This study was approved by the regional ethics board in Umeå (dnr 2017-249-31M). The Ethics board waived the need for written consent because the study was based on telephone interviews, and oral consent was given by all participants.

## Results

**Figure 2.**
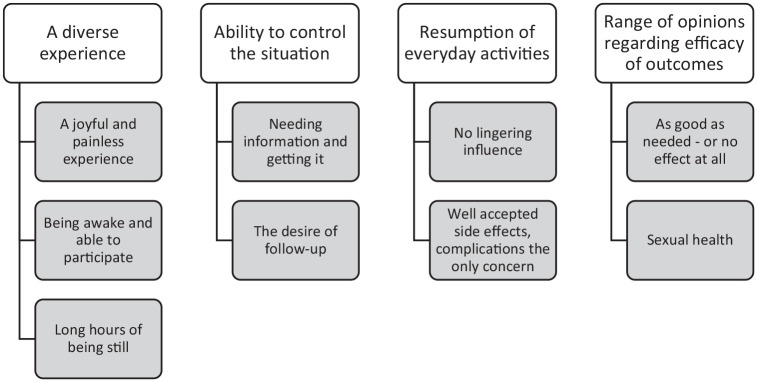
The results divided into categories and subcategories according to content analysis.

When discussing the PAE procedure, some focused more on the impact of the results while others focused on the actual procedure describing their joy or agony during the procedure. Participants’ experiences of PAE were formulated as four categories with subcategories as presented in Figure 2.

### A diverse experience

#### A joyful and painless experience

All participants agreed that the procedure was painlessness, saying they only had a sense of something happening in the region of the thigh and at most a sharp sting:

It was actually very pleasant and interesting, it was calm it did not hurt, and the staff was marvellous (ID014).

The warming sensation associated with administration of contrast made a few feel as if they had lost control over the bladder and bowel. A few participants experienced a chafing discomfort from the urinary catheter. Overall, the treatment was appreciated. Even those who did not benefit from the treatment would recommend the procedure to their friends, as it was easy and there were no long-lasting side effects.

#### Being awake and able to participate

Participants were interested in engaging in the procedure and enjoyed being able to ask questions and follow the process. To have a sense of what was happening inside their body was valued:

The operation was okay and rather fun to watch the doctor. (He) communicated all the time and told me what he was doing. I was able to watch them finding their way in the veins (ID013).

Participants expressed how the relaxed atmosphere in the theatre enhanced the positive feeling. However, a few patients would have rather been under general anaesthesia, mostly because of discomfort in being awake and exposed.

#### Long hours of being still

In theatre, the time was described as passing quickly, due to the interactive and friendly atmosphere. Some participants expressed how agonizing the procedure was, unable to adjust their painful position:

I lay entirely still, suffering from chafing on my scrotum, there was no one who thought about that, it was just accept the situation (ID002).

Ideas to improve the comfort were mentioned, a pillow to support the lumbar or being able to lie in a more upright position. Most trying was the 2 h of lying flat and still after the treatment with nothing to do.

### Ability to control the situation

#### Needing information and getting it

The need for information differed among the participants, especially prior to the procedure. More information on the mechanics of the procedure would have been appreciated by the participants. Curiosity surrounding the properties and abilities of the polyvinyl alcohol particles emerged and the desired level of detail varied:

The spheres would stick in there and it set me thinking on how and if they would cause any harm spreading further with the blood (ID012).

Despite a lack personal experience, the participants commonly viewed TURP as something unpleasant. Many had learned about PAE through media and had searched the Internet and discussed alternatives with their associates. Some had secondhand experience from fathers, friends or relatives. Most of the participants contacted their physician and presented the idea of PAE themselves.

The ability to recall the procedure varied, some had expressed a desire for more information but could still describe the procedure in detail. Others, happy with the given information, had more difficulty describing what had been done. Information post-treatment was satisfactory and a phone call from the surgeon a few days after the surgery was appreciated.

#### The desire of follow-up

Disappointment surrounding the later follow-up emerged, some participants expressed not knowing if, and when, it would occur. A few organized their own follow-up. The interest of knowing how their prostate might have changed post-treatment was communicated by several:

I still do not know and I still wonder if the prostate has shrunk at all [. . .] I do not know if they are going to examine it either (ID017).

### Resumption of everyday activities

#### No lingering impact

Prior to admittance, many organized the logistics surrounding their discharge, uncertain how they would feel afterwards. Most expressed these precautions were unnecessary:

I had no inconvenience after the treatment, on the contrary I was able to go out in the evening without noticing anything [. . .] I actually had arranged my nephew to pick me up by car and it was completely unnecessary (ID001).

Adhering to normal routines was not an issue, driving and carefully engaging in lighter work around the house the first days after treatment was common. A few patients described tiredness and feeling sore.

#### Well accepted side-effects, complications the only concern

Many did not experience any inconvenience, whereas others described painful micturition, lighter haematuria and sensations of tightness around the wound during the first weeks. These side-effects were tolerable and many of the participants would subject themselves to PAE again if they had to:

Initially it burned a bit but that gradually improved (ID020).

Participants who experienced relief would endure even greater side-effects to gain the outcome:

I would be willing to endure those side effects after treatment times 100 [. . .] to attain this improvement (ID014).

Complications mostly concerned malfunctioning urinary catchers, resulting in visits to the emergency department. Unexpectedly large bruises caused altered behaviour and unnecessary worry:

In addition to the bleeding I worried about a fairly large bruise which made me hesitate in taking a sauna bath (ID007).

### Range of opinions regarding efficacy of outcomes

#### As good as I need – or no effect at all

Some participants expressed a substantial improvement, others described a less dramatic effect in terms of regaining undisturbed sleep, less anxiety in social situations or a more relaxed attitude towards adventures where access to toilets might be a concern:

I feel more at ease I am not as afraid of having urine retention which bothered me especially when I was out traveling in Europe [. . .] I feel mentally stronger since I have no fear of anything happening (ID007).

Anticipating further improvement, curiosity towards redoing the procedure remained in the latter group, knowing others had experienced greater relief. Having had a pleasant experience in theatre, some lacked the desired results but regarded a theoretical possibility of less bleeding in case of a future TURP as positive:

I would say definitely, that others have nothing to lose. I feel the door is still wide open to do a TURP (ID010).

### Sexual health

Some participants addressed sexual health spontaneous, others were prompted. Sexual ability was of concern and a part of their decision when different methods were reviewed:

I function fully normally now, before the operation I had a lack of confidence (ID014).

Participants’ views of PAE’s impact on sexual health varied, some had regained libido and self-confidence. Others felt uncertain whether something had changed. A few participants expressed concern in lesser ability to keep erection but could not entirely associate this with the procedure.

## Discussion

In general, the participants have experienced PAE as a well-tolerated procedure. Comfort during treatment was perhaps the most important feature highlighted, as well as information prior to treatment. Areas suitable for improvement were discovered, for example, that a structured follow-up plan should be communicated prior to discharge. Because this method in many ways relates to percutaneous coronary intervention (PCI) and femoral artery angiography (FAA), it would be expected that patients’ experience would have been researched prior in the scope of these techniques. A few articles looking into aortic repair were found.^4,5^ The procedures are difficult to compare with PAE because of the life-threatening nature of aortic surgery and the largely different side-effects. Still a few similarities can be found, for example, that patients have a deep interest in their own health and body functions. Also, the need for information and a dialogue with health care professionals seem to be important. The results shown for PAE might be extrapolated to similar interventions with mild side-effects.

The joyful experience was mostly due to being awake and able to participate. A friendly environment during treatment was an important factor in how time and comfort was experienced by participants. In contrast, laying still with the vascular closing device (VSD) was described by many as very unpleasant. In accordance, a previous study on pain after surgery shows that severe pain during the postoperative phase is very common;^10^ compared with these results, PAE seems to be less painful than many other interventions. Participants suggested how to improve the comfort and entertainment to reduce the agony, being able to watch television or providing a short break to adjust the body position during the procedure. The performed surgeries were the first in Sweden, some were performed using a proctor, since then the skin-to-skin-time has been reduced, thus reducing the impact on the patient. The patient selection was not fully optimal because some of the patients had a lot of arteriosclerosis.

Information prior to treatment differed which might have been influenced by the influx of participants from different hospitals. Only some had the opportunity to discuss with the treating urologist/radiologist prior to accepting treatment. Many of the participants had spent some time and effort in finding an attractive treatment. The importance of patient education and thorough information has previously been highlighted in both breast reconstruction and emergency surgery,^7,11^ and access to information affects the perception of the quality of care.^12^ Studies prior to this have shown the importance of Internet when seeking health information^13^ also among elderly persons.^14^ Internet health information provides quality in terms of patient empowerment and ability to participate.^15,16^

Patients desire frequent follow-up.^17,18^ The participants expressed both a need to confirm the treatment outcome and an uncertainty regarding the persistence of the treatment effects. It is important to agree on the planned follow-up.

The participants did not suffer any adverse events post-treatment, and many resumed daily activities almost instantly. The degree of complications was as expected, shown in larger materials^19^ and well accepted among our participants. A study of femoral access for cardiac catheterization in a larger group of women showed low figures for postoperative pain.^20^

Participants’ experience of outcome efficacy differed. Some enjoyed remarkable results, and many were good enough. A few would have hoped for greater improvement. The success rate is lower for PAE compared to TURP,^21^ and there are concerns regarding the longevity.^22^ In this particular cohort, however, it is too early to assess the final outcome 1–12 months after PAE.

Limitations of the study include a potential risk of selection bias as the majority of participants enrolled themselves into the PAE procedure. Many participants researched alternatives to TURP before requesting treatment and this may have influenced the participants’ final opinion when asked to share their experience of undergoing PAE. The qualitative design does not provide data that can be presented using quantitative measures. To confirm to which extent a larger sample of PAE-patients would share the same views as the patients in this study, a questionnaire based on the results of the present study could be sent. The readability, accessibility and reliability of valid information regarding treatment alternatives also needs further investigation. If an identical study would be conducted with a more recent cohort with shorter time in the theatre, the results could possibly change a little bit with fewer of the patients complaining of agony of being still for example. The sample size included a majority of the men who had undergone PAE in Sweden as of September 2017 and saturation was reached. The study reflects the initial experience of PAE, early in the learning curve which could have affected the time in theatre and subsequently the level of associated discomfort.

## Conclusion

From the patients’ perspective, PAE is an appealing option for treating BPH. Viewed as painless and less invasive compared to TURP most would recommend the treatment to their friends. Self-enrolment may have influenced the results.

## Supplemental Material

sj-pdf-1-smo-10.1177_20503121211000908 – Supplemental material for Patients’ perspective on prostatic artery embolization: A qualitative study Click here for additional data file. Supplemental material, sj-pdf-1-smo-10.1177_20503121211000908 for Patients’ perspective on prostatic artery embolization: A qualitative study by Alexander Holm, Hans Lindgren, Mats Bläckberg, Marika Augutis, Peter Jakobsson, Mattias Tell, Jonas Wallinder, Karl-Johan Lundström and Johan Styrke in SAGE Open Medicine
