# Supplementary material for: Patients’ perspective on prostatic artery embolization: A qualitative study
Source: SAGE Open Med. 2021 Mar 12;9:20503121211000908. doi: 10.1177/20503121211000908 (PMC7958185; doi:10.1177/20503121211000908)
Supplement: sj-pdf-1-smo-10.1177_20503121211000908 – Supplemental material for Patients’ perspective on prostatic artery embolization: A qualitative study [file sj-pdf-1-smo-10.1177_20503121211000908.pdf]

Appendix 1 Semi structured Interview guide

What was your experience of the procedure?

How did you experience the preoperative information?

How did you experience lying in the theatre?

Did you feel sick during the operation?

Do you want to share anything else with me surrounding the operation?

How did you experience the time between the end of surgery and the time when you could return home?

How did you experience the week after the surgery?

Did you experience any side effects?

Did you experience sexual side effects?

Could you please describe the need for postoperative information?

Do you know if both prostate lobes were embolized?

Tell me about the current situation?

Weak urine flow?

Intermittency?

Urgency?

Incontinence?

Nocturia?

Could you please describe if your everyday life has changed after the procedure?

Can you tell me about the PAE-technique?

Did we miss any important question?

Would you recommend this to a friend and if so why?
